# Stability of Ampicillin plus Ceftriaxone Combined in Elastomeric Infusion Devices for Outpatient Parenteral Antimicrobial Therapy

**DOI:** 10.3390/antibiotics12030432

**Published:** 2023-02-22

**Authors:** Beatriz Fernández-Rubio, Laura Herrera-Hidalgo, Rafael Luque-Márquez, Arístides de Alarcón, Luis E. López-Cortés, Sonia Luque-Pardos, José María Gutiérrez-Urbón, Aurora Fernández-Polo, María V. Gil-Navarro, Alicia Gutiérrez-Valencia

**Affiliations:** 1 Unidad de Gestión Clínica de Farmacia, Hospital Universitario Virgen del Rocío, Instituto de Biomedicina de Sevilla (IBiS), 41013 Seville, Spain; 2 Unidad de Gestión Clinica de Enfermedades Infecciosas, Microbiología y Medicina Preventiva, Hospital Universitario Virgen del Rocío, Instituto de Biomedicina de Sevilla (IBiS), 41013 Seville, Spain; 3 Infectious Diseases and Microbiology Clinical Unit, University Hospital Virgen Macarena, Departament of Medicine, School of Medicine, University of Sevilla, Biomedicine Institute of Sevilla (IBiS)/CSIC, 41009 Seville, Spain; 4 Centro de Investigación en Red de Enfermedades Infecciosas (CIBERINFEC), Instituto de Salud Carlos III, 28029 Madrid, Spain; 5 Unidad de Gestión Clínica de Farmacia, Hospital del Mar, 08003 Barcelona, Spain; 6 Unidad de Gestión Clínica de Farmacia, Complexo Hospitalario Universitario de A Coruña, 15006 A Coruña, Spain; 7 Unidad de Gestión Clínica de Farmacia, Hospital Universitari Vall d’Hebron, 08035 Barcelona, Spain

**Keywords:** stability, outpatient parenteral antimicrobial therapy, elastomers, ampicillin, ceftriaxone, infective endocarditis

## Abstract

Currently, ampicillin plus ceftriaxone (AC) is one of the preferred treatments for *Enterococcus faecalis* infective endocarditis. However, there is a lack of stability data for the combination of both drugs in elastomeric devices, so the inclusion of AC in Outpatient Parenteral Antimicrobial Therapy (OPAT) programs is challenging. The objective of the study was to determine the stability of AC in elastomeric pumps when stored at 8 ± 2 °C, 25 ± 2 °C, 30 ± 2 °C and 37 ± 2 °C using LC-MS/MS. The combination was diluted in 0.9% sodium chloride and the final concentrations were ampicillin 24 g/L plus ceftriaxone 8 g/L. Physical and chemical stability were evaluated at 12, 20, 24, 36 and 48 h after preparation. Stability was met at each time point if the percentage of intact drug was ≥90% of its respective baseline concentration and color and clearness remained unchanged. The drug combination was stable for 48 h when it was kept at 8 ± 2 °C. At 25 ± 2 °C and 30 ± 2 °C, they were stable for 24 h of storage. At 37 ± 2 °C, the stability criterion was not met at any time point. These results prove that AC could be included in OPAT programs using elastomeric infusion devices for the treatment of *E. faecalis* infections.

## 1. Introduction

Infective endocarditis is a rare but life-threatening condition associated with high rates of morbidity and mortality. *Staphylococcus aureus* is the most prevalent cause of infective endocarditis, followed by viridans group streptococci, other streptococci and enterococci. Therefore, *Enterococcus* species are the third most common cause of infective endocarditis, excluding cases associated with injection drug use, and they represent around 10–15% of the total amount. *Enterococcus faecalis* is the leading agent of all enterococcal endocarditis, representing more than 90% of all cases, followed by *Enterococcus faecium* (5%) and other species [1]. Treatment of enterococcal endocarditis is known to be challenging due to the ability of enterococci to develop antibiotic resistance, with increasing high-level aminoglycoside resistance. Current American and European guidelines for the management of infective endocarditis caused by *E. faecalis* recommend dual beta-lactam therapy with ampicillin (AMP) and ceftriaxone (CRO) for 4–6 weeks as the first-line therapy [2,3]. After 2–3 weeks, most patients are clinically and hemodynamically stable, but they have to remain at hospital for another 3–4 weeks due to the antimicrobials’ intravenous administration [4]. In this context, Outpatient Parenteral Antimicrobial Therapy (OPAT) programs have been established as a high-quality and cost-effective alternative [5].

OPAT is a successful and safe care modality for patients who require intravenous treatment and have sufficient clinical stability to be at home [6]. Benefits of OPAT programs include a reduced length of hospital stay, readmission avoidance, a reduced risk of healthcare-associated infections, improved patient satisfaction and significant healthcare cost savings compared to inpatient care [7]. Outpatient clinic model, nurse home visits and self (or carer) administration are the OPAT modalities that have been explored [8]. Various drug administration strategies are safely employed in these programs, including infusion by gravity, portable programmable devices or elastomeric pumps. Elastomeric devices present numerous advantages, such as ease of management, since the manipulation of these devices only requires connection and disconnection from the catheter. Additionally, they are light, silent and do not require an external power supply for their functioning, allowing the complete mobility of the patient [9,10]. Among the limitations, the long-term use of elastomeric devices is more expensive than the one-time purchase of an electric infusion pump and the associated disposables, although continuous elastomeric pump infusion reduces the number of daily nurse visits [11,12]. Moreover, environmental factors such as the external temperature need to be taken into account, as they could affect the stability of the antimicrobials [13].

Consequently, the utilization of elastomeric pumps in OPAT programs for the treatment of *E. faecalis* infective endocarditis appears to be valuable and useful [14]. At the hospital, the standard dosage of AMP and CRO used is 2 g every 4 h and 2 g every 12 h to treat this infection, respectively. In the OPAT setting, continuous administration through elastomeric devices could be an interesting alternative, given the fact that both antimicrobials are beta-lactams, so continuous infusion would achieve higher plasma antibiotic concentrations than intermittent administration and would improve the clinical cure. Our study group has already proven that the combination of both antimicrobials at the same daily dose (12 g of AMP and 4 g of CRO per day) is stable for up to 30 h at 25 °C and 30 °C when they are diluted in 0.9% sodium chloride polypropylene infusion bags [15]. Therefore, it is no longer necessary to use two simultaneous electronic devices, since daily doses of both antibiotics can be dissolved in the same solution and they can be administered through a single electronic pump. Nevertheless, it was demonstrated that the storage container is a factor that can have an impact in drug absorption, so it is necessary to study the stability data for this antimicrobial combination in elastomeric devices [16]. Additionally, the combination of AMP plus CRO could also be suitable in other infections, such as osteomyelitis caused by *E. faecalis*, or even against other microorganisms such as *Listeria monocytogenes* in invasive infections, especially when the central nervous system is compromised [17,18].

The purpose of the present study was to determine the stability of AMP plus CRO in elastomeric pumps when stored under refrigerated conditions and at three different room temperatures. This information will provide guidance regarding the stability and the potential use of this combination for the treatment of *E. faecalis* infective endocarditis in OPAT programs.

## 2. Materials and Methods

**Materials.** Ampicillin sodium was obtained from Normon Laboratories (Madrid, Spain) and ceftriaxone sodium for injection was purchased from Medochemie Limited (Limassol, Cyprus). The internal standard cefixime was supplied by Alsachim (Illkirch, France). Dosi-fuser elastomeric pumps and tube clamps were supplied by Leventon (Barcelona, Spain). Normal saline 0.9% sodium chloride bags were purchased from Baxter Healthcare, Inc., Deerfield, Illinois. Liquid chromatography–mass spectrometry (LC-MS)-grade (reagent-grade, > 98% pure) acetonitrile was obtained from Merck KGaA (Darmstadt, Germany), and formic acid was purchased from Scharlab (Barcelona, Spain). Ammonium acetate was obtained from Fisher Scientific (Newington, NH, USA). Purified water was prepared in-house with a Milli-Q water system from Millipore (Bedford, MA, USA).

**Preparation of solutions.** AMP and CRO were reconstituted with water for injection (10 mL per each gram of antibiotic), resulting in a concentration of 100 g/L. According to the current guidelines for the treatment of endocarditis caused by *E. faecalis*, the daily dosages of AMP and CRO (12 g and 4 g, respectively) were diluted together in 0.5 L of 0.9% sodium chloride and introduced into the polyisoprene elastomeric pumps. Therefore, the final concentrations obtained were AMP 24 g/L and CRO 8 g/L. Three different elastomeric pumps were prepared for each temperature condition.

**Storage conditions.** Antibiotic stability was tested at 8 ± 2 °C, 25 ± 2 °C, 30 ± 2 °C and 37 ± 2 °C. Once initial (0 h) samples were collected, the elastomeric pumps were stored in an air thermostat oven for the duration of the study (48 h). In-line filters and flow restrictors were removed from the elastomeric devices for the study and the outlet was clamped using a suitable tube clamp. At each time point (0, 12, 20, 24, 36 and 48 h), duplicate 1 mL samples were taken and frozen at −80 °C until the analysis.

**Sample processing.** Before the analysis, samples were diluted in Milli-Q water, vortexed and aliquoted in autosampler vials. Five microliters of this solution were injected into the HPLC-MS/MS device. The color and clarity of each elastomeric pump solution were visually assessed at each sampling time point.

**LC-MS/MS quantification**. Antibiotics concentrations were measured by a liquid chromatography–tandem mass spectrometry (LC-MS/MS) assay using a method previously described [15]. Validation of the method was performed following FDA guidelines, and the results met the acceptance criteria [19].

Briefly, samples were analyzed by using an Agilent 1290 Infinity liquid chromatograph (Agilent Technologies, Palo Alto, CA) equipped with a 1260 Bin Pump VL binary pump and a HiP-ALS autosampler pump coupled to an AB SCIEX API 4000 mass spectrometer (AB Sciex, Darmstadt, Germany) operating in electrospray positive ionization mode. Monitored transitions were 350.178 → 106.100 (AMP), 555.1 → 359.9 (CRO) and 454.0 → 285.0 (cefixime, internal standard) *m/z*. The column and auto-sampler tray temperatures were set at 40 °C and 4 °C, respectively. Separation was performed on a Phenomenex Luna C18 analytical column (5 mcm, 150 × 2.0 mm) with isocratic elution (70/30). The mobile phase consisted of 10 mM ammonium acetate and 1% formic acid in Milli-Q water (phase A) and 0.1% formic acid in acetonitrile (phase B). Ten microliters were injected into the column and both drugs were eluted at a flow rate set at 0.5 mL/min.

**Chemical stability**. Drug stability was calculated as the percentage (*P*) of the initial drug remaining in the elastomeric device at each analyzed time point (*Ct*), in relation to the baseline concentration (*C*0) (*P* = *Ct/C*0 × 100). Stability was met at each time point if the mean of the triplicate assays contained ≥ 90% of its respective baseline concentration [16]. Data are expressed as means and their 90% confidence intervals (CIs).

**Physical stability.** Precipitation, transparency and color of each elastomeric pump were visually assessed at each sampling time point. For the evaluation of the appearance of the solutions, the data were evaluated based on “no significant change” from the initial time point solution to the hold solutions.

## 3. Results

**Chemical stability**. Percentages and 90% CIs of the remaining concentrations that were observed at each analytic time point during 2 days for each storage condition are listed in Table 1.

At refrigerated temperature (8 ± 2 °C), AMP and CRO attained the stability criterion of >90% of the original concentration for the whole experiment. On the contrary, at 25 ± 2 °C, ampicillin attained the stability criterion of >90% until 24 h of storage. At the same temperature, CRO remained stable after 48 h of storage in the elastomeric infusion device. At 30 ± 2 °C, AMP was stable for 24 h and CRO attained the stability criterion of >90% of the original concentration for 36 h. Before 12 h at 37 ± 2 °C, both concentrations of AMP and CRO dropped below 90%.

**Figure 1:**
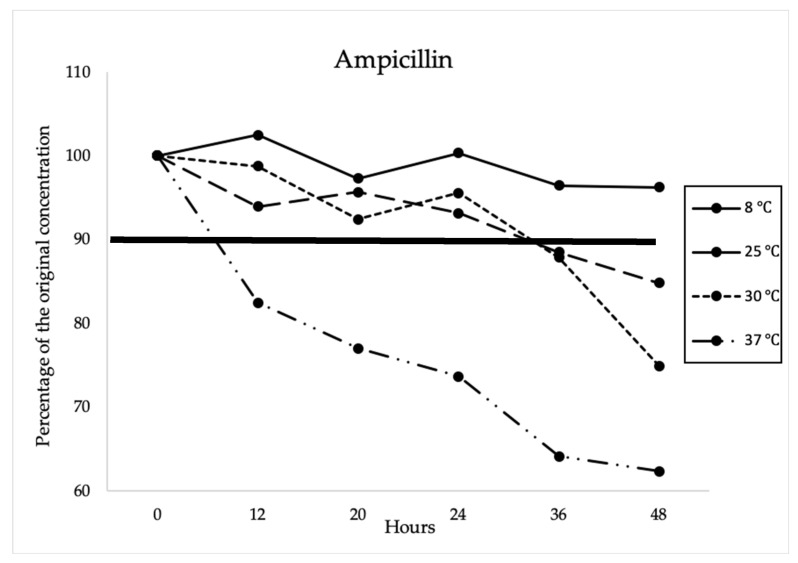
AMP stability expressed as mean percentage of original concentration over time at 8 ± 2 °C, 25 ± 2 °C, 30 ± 2 °C and 37 ± 2 °C. Dashed line indicates 90% of residual ratio.

**Figure 2:**
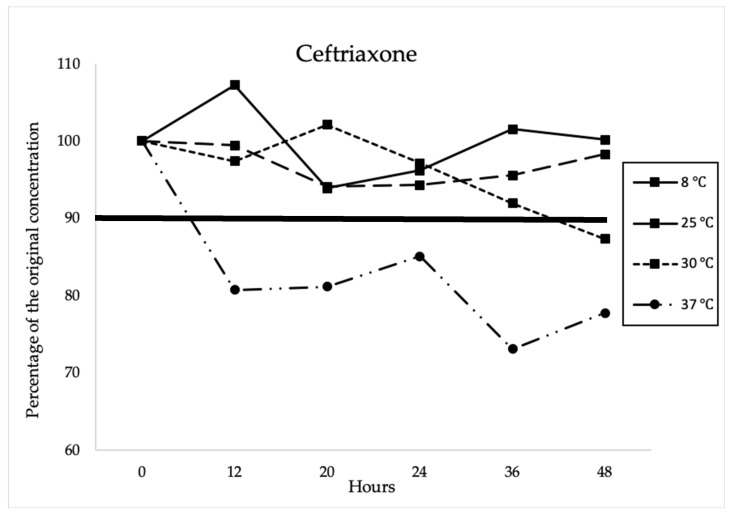
CRO stability expressed as mean percentage of original concentration over time at 8 ± 2 °C, 25 ± 2 °C, 30 ± 2 °C and 37 ± 2 °C. Dashed line indicates 90% of residual ratio.

AMP and CRO solution stability data are depicted in Figure 1 and Figure 2, respectively.

**Physical stability**. There were no observed changes in clarity or color and no visible precipitation appeared in any sample during the whole experiment (48 h).

## 4. Discussion

The present study has evidenced that AC combined in elastomeric devices could be included in OPAT programs, although the external temperature needs to be controlled: in warmer environments, where 37 °C can be reached, the combination of both drugs should not be used, since its concentration drops below 90% in the first 12 h after its preparation. Nonetheless, temperatures between 25 °C and 30 °C are more common in elastomeric infusion devices, and we have demonstrated the stability of AC at both conditions until 24 h of storage [20]. Additionally, both drugs attained the stability criterion of >90% of the original concentration for 48 h at refrigerated temperatures.

Currently, AMP 2 g every 4 h combined with CRO 2 g every 12 h is one of the recommended therapies for the treatment of *E. faecalis* infective endocarditis [2,3]. This double beta-lactam combination has been supported by preclinical data proving its synergistic activity and clinically good results in many cohorts [21,22,23]. However, the administration of this regimen via OPAT is complex due to the requirement of two venous accesses and the need for the twice-daily administration of CRO, since its delivery in a single daily dose of 4 g has been associated with insufficient drug concentrations throughout the entire administration interval, with a greater number of relapses reported in a clinical cohort [24]. For decades, AMP plus gentamycin was the first election, even though it was set aside due to the high rate of toxicity, especially in frail elderly patients [25,26]. Different alternatives for the treatment of *E. faecalis* infective endocarditis suitable in the outpatient setting have been proposed, such as teicoplanin or dalbavancin through OPAT [27,28]. In the first case, the main advantage is its long elimination half-life, which permits a once-daily dose, and it is not associated with renal toxicity [27]. Regarding dalbavancin, it is approved in the USA and Europe to treat adults with skin infections caused by several Gram-positive cocci isolates, including vancomycin-susceptible *E. faecalis*. One of the most important benefits of dalbavancin is its exceptional pharmacokinetic profile, which allows its administration every several days. However, the available evidence for the treatment of *E. faecalis* infective endocarditis in both cases is still limited, so further studies are needed [28]. In the last few years, new alternatives such as daptomycin or oral treatment have emerged [29,30]. Daptomycin is a cyclic lipopeptide antibiotic with highly concentration-dependent bactericidal activity against Gram-positive bacteria and has been used in the setting of resistant enterococci. However, the conclusions obtained in the studies that assessed this therapeutic approach in *E. faecalis* infective endocarditis were radically different, so more investigations are needed before clear recommendations can be made [29]. With regard to oral antibiotic treatment, it seems attractive since it avoids the need for intravenous catheters. Moreover, it shows normal gastrointestinal uptake and good adherence. Amoxicillin-based regimens at high doses and linezolid are the two oral therapies that have been explored, but, in the two cases, further studies are needed to safely decide which patients could complete their antibiotic treatment using oral alternatives [30].

To our knowledge, the stability of AC combined in elastomeric devices has not been previously studied. Nevertheless, several articles have reported data about the stability of both antibiotics separately in elastomeric pumps [31,32,33,34]. Regarding AMP, the conditions for each measurement were different in terms of the elastomer composition (latex, silicon or polyisoprene), concentration (20, 30 or 50 g/L) and diluent (0.9% sodium chloride, sterile water or acetate ringer solution) [31,32,33]. Therefore, the reported results are considerably different: at 20 g/L using 0.9% sodium chloride as a diluent and latex as the material of the elastomeric reservoir, AMP was chemically stable for 3 days when it was stored refrigerated for 8 h at 25 °C [31]. When the concentration was 30 g/L using a silicon-based elastomeric system, AMP preserved > 90% of the initial concentration after 3 days stored at 25 °C in 0.9% sodium chloride and 2 days in sterile water [32]. However, it was chemically stable for 7 days using both diluents at refrigerated temperatures. At the highest concentration, 50 g/L, diluted in acetate ringer solution and contained in a polyisoprene reservoir, AMP was chemically unstable at 25 °C and 31.1 °C, probably due to its high concentration [33]. In relation to CRO’s chemical stability in elastomeric pumps, two studies have been carried out [31,34]. The first one found it to be stable at 20 g/L for 10 days at refrigerated temperatures and 3 days at 25 °C in a latex reservoir using 0.9% sodium chloride or 5% dextrose as diluents [31]. Similarly, at a concentration of 5 g/L and 40 g/L diluted in 0.9% sodium chloride or 5% dextrose solution and contained in silicon, the stability was 14 days at 4 °C and 2 days at room temperature [34]. The stability of CRO in elastomeric devices at temperatures above 30 °C had not been assessed prior to this study.

Thus, our data provide sufficient evidence to allow the administration of AC combined through an elastomeric pump when the temperature is under 30 °C for 24 h. Only two previous investigations studied the stability of AC combined, although both the methodology and the results were remarkably different from our study [15,35]. In the first case, it was a simulation of Y-site administration using glass test tubes over 4 h, with uncertain results [33]. Regarding the other investigation, it was carried out by our research group but using a different infusion device, polypropylene infusion bags, and it was widely demonstrated that the composition of the delivery devices, as well as the antibiotic concentration and temperature, were significant modifiers of the stability of drugs [15,16]. Consequently, this study shows the stability of AC combined in elastomeric infusers at the usual conditions of OPAT programs, providing essential information to enhance its use. Given the fact that our results corroborate the safety of AC combined in terms of drug stability for 48 h at refrigerated temperatures and for 24 h at 25 °C and 30 °C, the mixture can be administered through continuous infusion at home. Therefore, patients who are clinically stable can be discharged and complete their treatment at home using elastomeric pumps. In addition, as the elastomeric devices need to be replaced only once a day by a nurse or by the patient himself, it avoids multiple daily interventions, so there is a significant improvement in the quality of life of the patient [36]. Moreover, it is established that the pharmacodynamic parameter best related to AMP activity, as with the rest of the beta-lactam antibiotics, is the time above the MIC (T > MIC), so prolonged plasma concentrations are necessary for an optimal treatment. AMP is poorly bound to plasma protein (10%) and has a half-life of 2 to 4 h, and this is the reason that it usually requires multiple and frequent administrations, which can be avoided by using continuous infusion [37]. Furthermore, considering the refrigerated stability proven in this study, 48 h, it seems reasonable to prepare simultaneously two elastomers, one for immediate use at a maximum temperature of 30 °C, and the other to be stored refrigerated for 24 h until usage. This strategy would improve OPAT resource management, since it could reduce costs associated with nursing visits and pharmacy drug preparation to every two days. Nonetheless, before general recommendations are made, further studies are required to prove the stability of both antibiotics after sequential storage at two temperatures—for example, 24 h at refrigerated temperatures, followed by another 24 h at 25 °C or 30 °C.

In order to further improve the quality of life of patients included in OPAT programs, the utilization of elastomeric pumps is growing. However, it has been demonstrated that the temperature reached in these devices may increase much higher than 25 °C due to direct exposure to sunlight [38,39]. Additionally, when the pump stays in direct contact with the patient’s body—for example, around the waist—and is kept under a blanket during nighttime, the temperature may rise up to 32 °C. Moreover, in warmer climates, the solution temperature might occasionally reach temperatures around 38 °C [40]. In this scenario, the National Health System (NHS) in the UK established a document named the “Standard Protocol for Deriving and Assessment of Stability”, commonly known as the Yellow-Covered Document (YCD), that describes the minimum data required from a study to be applicable for use in the OPAT context [41]. In this document, warm temperature data are considered essential. In the same way, a recent systematic review concluded that there is an urgent need for studies that verify the stability and appropriate use of many antibiotics used in OPAT at standard room temperature and in warmer climates [42].

Among the strengths that can be found in our study, it is remarkable that we included a range of temperatures similar to those achievable due to real-life temperature variations at home. Regarding the technique employed, we used HPLC coupled to tandem mass spectrometry (MS/MS), which, compared to other detectors, such as ultraviolet, is more robust, sensitive and specific [43]. Lastly, the composition of the elastomeric devices that we tested in our study included polyisoprene, the most commonly material used in the elastomeric pumps applied in OPAT programs [44]. Our study has also some limitations: although 90% chemical stability has been established as defined in the European and US Pharmacopoeias, recently, a 95–105% limit has been proposed [19,41]. Secondly, pH variations were not measured. However, since pH changes are related to AMP degradation and its concentrations were measured using LC-MS/MS, we did not consider it necessary [45,46]. In the third place, the effect of additives on the stability has not been investigated [47,48]. Despite the fact that each generic or name-brand drug may not contain the same additives, the number of brands available in the market made it unfeasible to test them all. Impurities or others degradation products were not measured either.

## 5. Conclusions

In summary, the importance of the present study resides in the possibility of including AC in OPAT programs using elastomeric infusion devices for the treatment of *E. faecalis* infections. This drug combination is stable for 48 h when it is kept at 8 ± 2 °C and it can be used for 24 h if the temperature reached is 25 ± 2 °C or 30 ± 2 °C. However, at 37 ± 2 °C, the mixture should not be used, since it becomes unstable in the first 12 h after its preparation.

## Figures and Tables

**Table 1:** Stability at 8 ± 2 °C, 25 ± 2 °C, 30 ± 2 °C and 37 ± 2 °C.

Temperature	Antibiotic	Concentration Remaining (90% CI)				
		12 h	20 h	24 h	36 h	48 h
8 ± 2 °C	AMP	102.50 (106.62–98.37)	97.32 (103.96–90.68)	100.32 (105.30–95.35)	96.46 (100.12–92.79)	96.23 (99.07–93.40)
	CRO	107.27 (112.00–102.53)	93.88 (97.44–90.32)	96.19 (99.81–92.57)	101.52 (106.89–96.14)	100.17 (106.13–94.21)
25 ± 2 °C	AMP	93.96 (97.82–90.09)	95.67 (100.26–91.08)	93.17 (95.15–91.19)	88.52 (90.42–86.62)	84.81 (88.03–81.59)
	CRO	99.46 (102.39–96.52)	94.11 (97.26–90.97)	94.31 (96.05–92.56)	95.59 (99.65–91.53)	98.28 (104.49–92.06)
30 ± 2 °C	AMP	98.75 (102.91–94.58)	92.44 (94.87–90.01)	95.59 (99.86–91.33)	87.89 (92.01–83.76)	74.96 (75.33–74.58)
	CRO	97.41 (104.18–90.64)	102.14 (109.35–94.93)	97.16 (102.19–92.14)	91.97 (93.79–90.16)	87.36 (91.06–83.65)
37 ± 2 °C	AMP	82.49 (85.40–79.59)	77.05 (80.41–73.69)	73.66 (77.41–69.92)	64.16 (67.70–60.62)	62.35 (65.28–59.41)
	CRO	80.72 (85.55–75.90)	81.14 (85.73–76.56)	85.07 (89.60–80.54)	73.11 (76.11–70.11)	77.78 (81.47–74.09)

## Data Availability

Not applicable.
